# Predictive value of TGF-β1 and SMAD-7 expression at diagnosis for treatment response in low-risk myelodysplastic syndrome

**DOI:** 10.17305/bb.2025.11564

**Published:** 2025-03-03

**Authors:** Bedrettin Orhan, Hülya Öztürk Nazlıoğlu, Oğuzhan Dik, Büşra Gürbüz, Vildan Özkocaman, Tuba Ersal, İbrahim Ethem Pınar, Cumali Yalçın, Sinem Çubukçu, Tuba Güllü Koca, Fazıl Çağrı Hunutlu, Şeyma Yavuz, Rıdvan Ali, Fahir Özkalemkaş

**Affiliations:** 1 Division of Hematology, Department of Internal Medicine, Faculty of Medicine, Bursa Uludağ University, Bursa, Türkiye; 2 Department of Pathology, Faculty of Medicine, Bursa Uludağ University, Bursa, Türkiye; 3 Department of Internal Medicine, Faculty of Medicine, Bursa Uludağ University, Bursa, Türkiye

**Keywords:** Myelodysplastic syndrome, MDS, Mothers against decapentaplegic homolog 7, SMAD-7, transforming growth factor beta 1, TGF-β1, erythropoietin stimulating agent, ESA

## Abstract

Myelodysplastic syndrome (MDS) is a clonal hematopoietic stem cell disease. Supportive treatments, such as erythropoiesis-stimulating agents (ESAs), are commonly used in patients with low-risk MDS. This study aimed to retrospectively assess the impact of bone marrow Mothers against decapentaplegic homolog 7 (SMAD-7) and transforming growth factor beta 1 (TGF-β1) protein expression on prognosis and response to ESA treatment in patients with low-risk MDS. We retrospectively analyzed patients diagnosed with low-risk MDS at the adult hematology department of Bursa Uludağ University Hospital. A total of 56 patients classified as low or very low risk were included in the study. Immunohistochemical analysis of bone marrow specimens at diagnosis showed that only five patients (9.8%) exhibited low SMAD-7 staining, while 51 patients (90.2%) showed no staining. Regarding TGF-β1 staining, 18 patients (32.1%) demonstrated moderate to high staining, whereas 38 patients (67.9%) exhibited low (36/38) or no staining (2/38). A statistically significant correlation was found between TGF-β1 staining levels and ESA treatment administration (*P* ═ 0.011). Additionally, a significant relationship was observed between lower erythropoietin (EPO) levels and moderate to high TGF-β1 staining (*P* ═ 0.04). However, when TGF-β1 staining status was compared with first- and third-month treatment responses in patients receiving ESA therapy, no significant difference was detected between groups. These findings suggest that while TGF-β1 alone may not be sufficient to predict ESA treatment response, additional parameters related to the TGF-β/SMAD pathway should be considered. Strong TGF-β1 staining, alongside EPO levels, may influence the decision to initiate ESA therapy.

## Introduction

**Figure 1.**
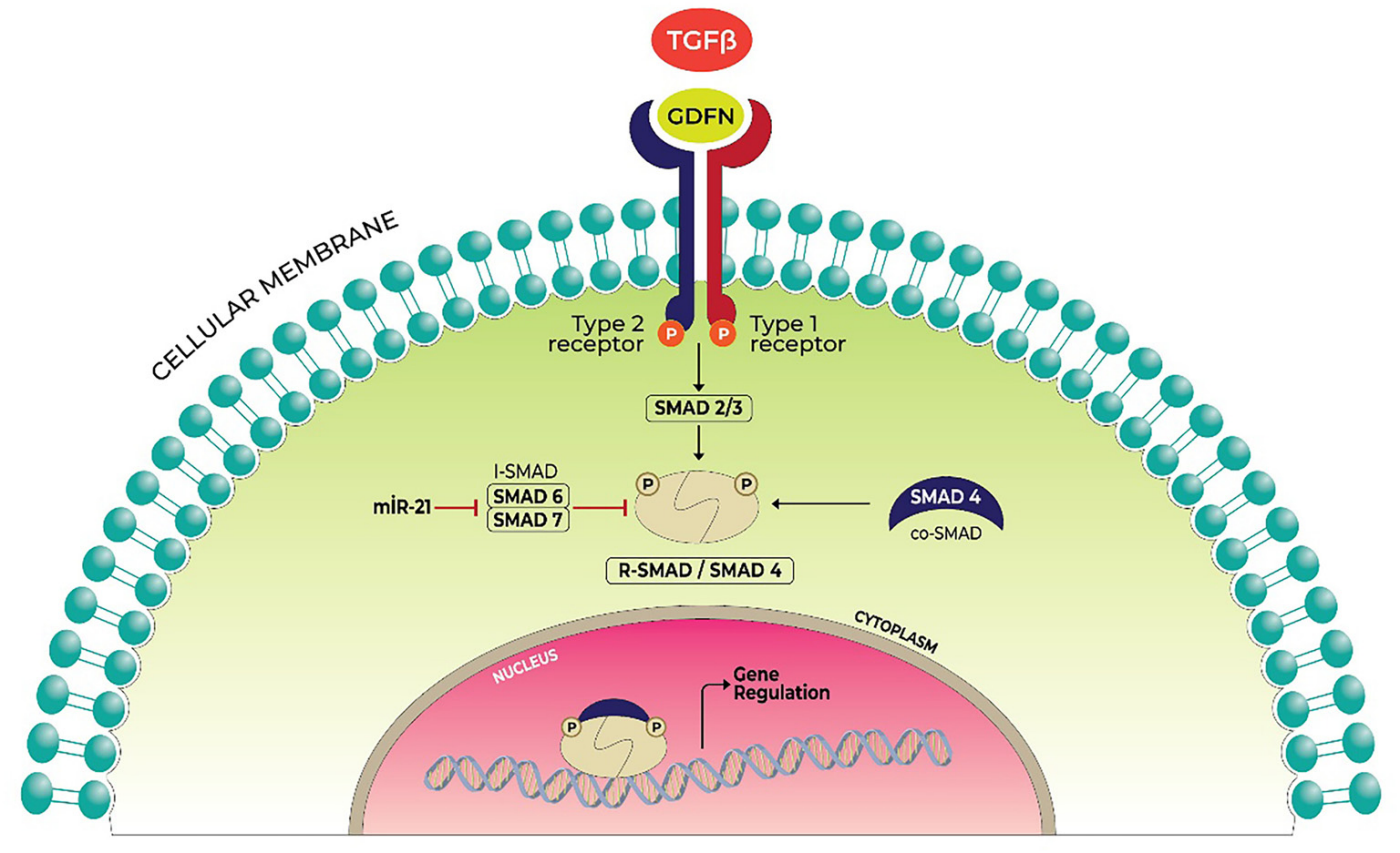
**Relation between TGF-beta-1 and SMAD proteins.** Inspired and re-illustrated from Parisi et al. [8]. TGF: Transforming growth factor; SMAD-7: Mothers against decapentaplegic homolog 7.

Myelodysplastic syndrome (MDS) is a clonal hematopoietic stem cell disease characterized by varying degrees of cytopenias and morphological dysplasia due to ineffective hematopoiesis, with a risk of transformation into acute myeloid leukemia (AML) [1]. Given the heterogeneity in symptom burden and AML risk, treatment selection is typically individualized using tools like the Revised International Prognostic Scoring System (IPSS-R) [2–4]. The IPSS-R classifies patients based on cytopenias, bone marrow blast percentage, and cytogenetic risk groups. While intermediate- and high-risk MDS patients often receive aggressive treatments such as hypomethylating agents or allogeneic stem cell transplantation, supportive therapies—including blood transfusions, iron chelation, erythropoiesis-stimulating agents (ESAs), granulocyte colony-stimulating factor (G-CSF), and thrombopoietin—may be sufficient for low-risk patients [5, 6]. Cytokines play a crucial role in regulating normal hematopoiesis, and a balance between hematopoietic growth factors and myelosuppressive factors is essential for optimal blood cell production [7]. In MDS, overproduction of inhibitory cytokines exacerbates ineffective hematopoiesis [1]. Transforming growth factor-beta (TGF-β), a myelosuppressive cytokine, is particularly implicated in hematopoietic suppression. TGF-β signaling induces apoptosis and cell cycle arrest in erythroblasts, inhibiting erythroid differentiation [8]. Additionally, elevated TGF-β levels have been linked to fibrosis and malignancies in various tissues and organs [9–11]. The TGF-β signaling pathway is regulated by both inhibitory (SMAD-6/7) and activating (SMAD-2/3) SMAD proteins (Suppressor of Mothers against Decapentaplegic [SMAD]). These intracellular proteins transmit signals from extracellular TGF-β1 receptors to the nucleus, where they initiate gene transcription. Among them, Mothers against decapentaplegic homolog 7 (SMAD-7) serves as a key negative regulator in the TGF-β1/SMAD-7 signaling pathway [1]. TGF-β stimulates SMAD-2/3 and SMAD-4, triggering changes in target gene expression in the nucleus. This pathway is tightly regulated by SMAD-6 and SMAD-7, which act as inhibitors. The levels of these inhibitory SMAD proteins, particularly SMAD-7, are influenced by microRNA-21 (Figure 1). Notably, microRNA-21 levels are elevated in the bone marrow cells of patients with MDSs compared to age-matched healthy controls, leading to SMAD-7 inhibition [8]. Consequently, SMAD-7 expression—normally a negative regulator of the TGF-β1 receptor—is significantly reduced in MDS patients [7]. Studies indicate that suppressed SMAD-7 protein levels in MDS patients can be reactivated by certain substances. When SMAD-7 is reactivated, TGF-β levels decrease, thereby alleviating hematopoietic suppression [1, 2]. For patients with low-risk MDS, erythrocyte suspensions and ESA therapy can improve quality of life. However, these treatments also pose long-term cardiovascular risks due to chronic anemia and increased iron accumulation [12]. Additionally, some patients exhibit high baseline erythropoietin (EPO) levels, which reduces their responsiveness to exogenous ESA therapy. Given that ineffective erythropoiesis and apoptosis in MDS are primarily driven by dysregulation in the TGF-β signaling pathway, therapies targeting this pathway offer promising potential [13, 14]. Treatment options remain limited for low-risk, transfusion-dependent MDS patients who either do not respond to ESAs or have pretreatment EPO levels >500 U/L. Luspatercept, an activin receptor IIB-immunoglobulin G (IgG) Fc-fusion protein, enhances erythropoiesis by binding to growth differentiation factor 11 (GDF-11) and modulating SMAD-2/3 and TGF-β signaling. Clinical studies have shown that luspatercept effectively reduces transfusion frequency or leads to transfusion independence, particularly in MDS patients with ring sideroblasts or those with high basal EPO levels [15]. Recently, the FDA approved luspatercept for the treatment of patients with MDS-ring sideroblasts (MDS-RS) [16]. Although well-defined predictive scores, such as the MDS-CAN ESA and ITACA scores, indicate ESA treatment response in low-risk MDS patients, these scores do not incorporate immunohistochemical studies [17, 18]. Given this limitation, there is growing interest in the potential role of the TGF-β/SMAD pathway in inducing hematopoiesis in MDS patients. To the best of our knowledge, no studies have explored the relationship between TGF-β1 expression and ESA treatment response. Therefore, this study aimed to retrospectively assess the impact of bone marrow SMAD-7 and TGF-β1 protein expression at diagnosis on prognosis and ESA treatment response in patients with low-risk MDS.

## Materials and methods

### Patient selection and parameters evaluated

Patients with low-risk MDS who were examined by the adult hematology department at Bursa Uludağ University Hospital between 2016 and 2021 were retrospectively analyzed. Due to concerns about antigenic loss, as described by Hayat [19], preparations dated before 2016 were excluded from the study. Additionally, because of the country’s health policies during the study period, ESA treatment could not be initiated in patients with EPO levels below 500. Patients were included in the study if their EPO levels were measured at the time of diagnosis, their bone marrow preparations were available at our center, their preparations were suitable for paraffin blocks, and they were classified as low or very low risk according to the R-IPSS classification. The study evaluated patients’ median age, gender, and laboratory parameters at the time of diagnosis (hemoglobin, neutrophils, lymphocytes, monocytes, platelets, and EPO). It also assessed whether they received ESA treatment (darbepoetin 100 mcg/week or epoetin alpha 150 IU/kg), whether they used G-CSF during follow-up, comorbidities, their first- and third-month responses to ESA treatment, iron chelation status, secondary AML progression, and mortality status. Cytogenetic anomalies, including del (5q), del (20q), del (11q), –7, del (7q), +8, and translocations t (8:21), t (15:17), t (9:22), t (12:21), and inv (16), were evaluated. Control bone marrow biopsies were performed when there was clinical suspicion of AML transformation. Patients’ comorbid conditions were examined to determine whether hepatic and endocrine diseases, which have been linked to the TGF-β1/SMAD-7 pathway in the literature [9, 10], would influence the study results. Progression was defined as the time from diagnosis to secondary AML. Bone marrow TGF-β1 and SMAD-7 staining at the time of diagnosis was evaluated by two pathologists. SMAD-7 staining was categorized as either no staining or weak staining, in accordance with the literature [20]. TGF-β1 staining was classified as no/weak staining or moderate/strong staining, based on both literature findings and statistical significance [21].

### Immunohistochemical staining

**Figure 2.**
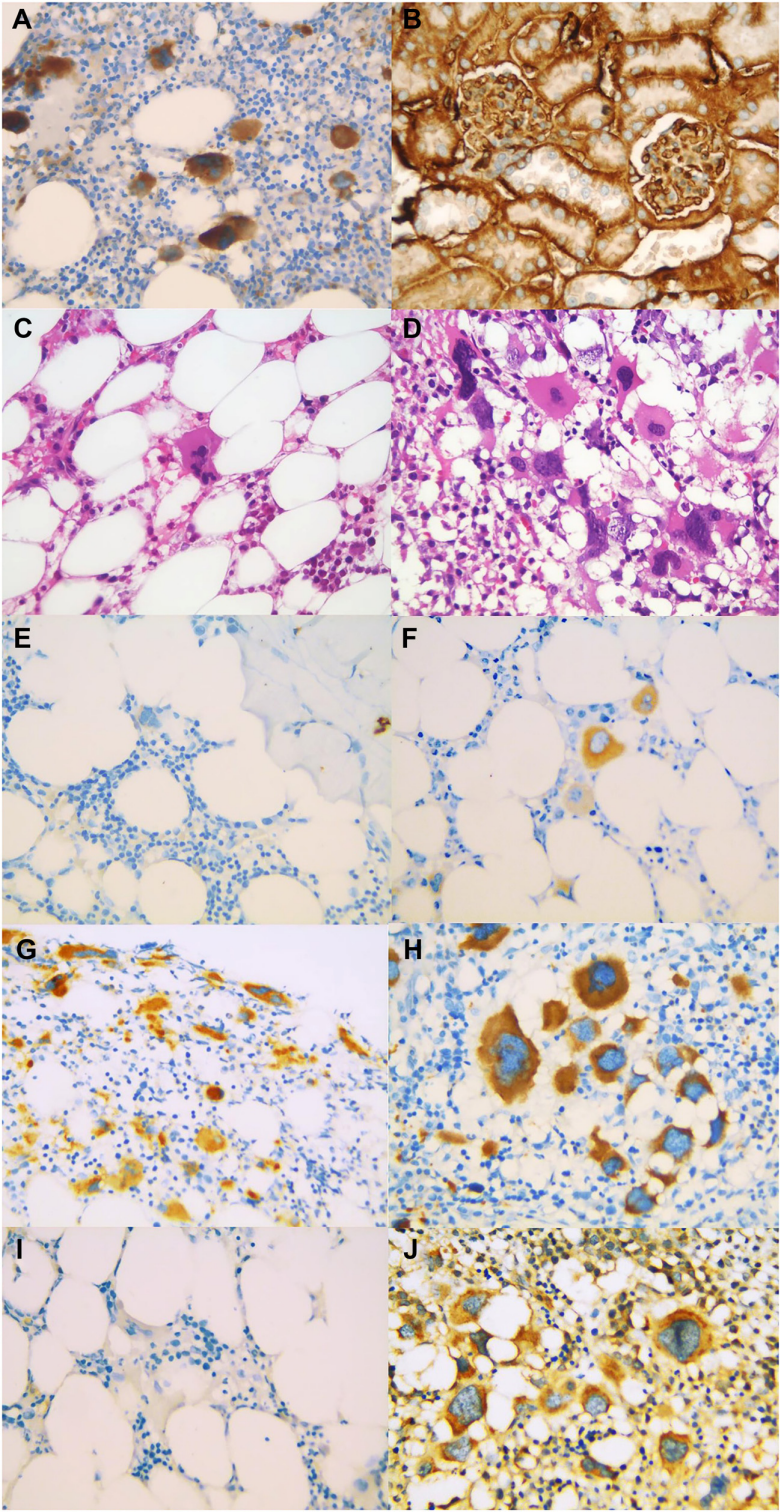
**Control groups 1 × 400 magnification**. (A) TGF-β1, human bone marrow tissue, red arrows showing megakaryocytes; (B) SMAD-7, mouse kidney tissue; (C) Hematoxylin and eosin slides for SMAD-7; (D) Hematoxylin and eosin slides for TGF-β1. Bone marrow tissue TGF-β1 staining, showing stained megakaryocytes; (E) No staining, 1 × 400 magnification; (F) Weak staining, 1 × 400 magnification; (G) Moderate staining, 1 × 400 magnification; (H) Strong staining, 1 × 400 magnification. SMAD-7 staining and megakaryocyte staining of bone marrow tissue; (I) No staining 1 × 400 magnification; (J) Weak staining 1 × 400 magnification. TGF- β1: Transforming growth factor beta 1; SMAD-7: Mothers against decapentaplegic homolog 7.

Paraffin sections (4 µm thick) were baked overnight at 50 ^∘^C. Deparaffinization was performed using the Ventana Discovery XT platform with EZ Prep solution (Roche, Ventana Medical Systems, Tucson, AZ, USA; catalog number 950–100) at 75 ^∘^C for 8 min. Standard antigen retrieval was conducted via Heat-Induced Epitope Retrieval in Tris-EDTA buffer (pH 7.8) at 95 ^∘^C for 64 min, using standard cell conditioning solution (CC1) (Roche, Ventana Medical Systems, Tucson, AZ, USA; catalog number 950–124) on the Ventana Discovery XT platform. To block endogenous peroxidase and proteins, inhibitor CM was applied at 37 ^∘^C for 4 min using the Ventana Discovery XT. The primary antibodies used were TGF-β1 (1:300 dilution, Abcam, USA; catalog number ab215715) and SMAD-7 (1:100 dilution, Abcam, USA; catalog number ab216428). The TGF-β1 primary antibody was incubated at 37 ^∘^C for 36 min, while the SMAD-7 primary antibody was incubated at 37 ^∘^C for 60 min. For controls, mouse kidney tissue was used for SMAD-7, and a bone marrow specimen from a patient with essential thrombocythemia was used for TGF-β1 (Figure 2). Detection was performed using the ultraView Universal DAB Detection Kit (Roche, Ventana Medical Systems, Tucson, AZ, USA). Finally, tumor specimens were examined under a light microscope (model BX51TF, Olympus, Tokyo, Japan).

### Ethical statement

Our study was conducted by the institutional research committee’s ethical standards and the 1964 Helsinki Declaration. This study was approved by the clinical research ethics committee of the Bursa Uludağ University Faculty of Medicine (Decision No: 2021-6/21).

### Statistical analysis

Statistical analyses were conducted using IBM SPSS Statistics for Windows, Version 25.0 (IBM Corp., Armonk, NY, USA). Descriptive statistics were reported as *n* (%) for categorical variables and as mean ± SD or median (min–max) for continuous variables. Normality assumptions were assessed, and based on the distribution of variables, either the independent *t*-test (for parametric data) or the Mann–Whitney *U*-test (for non-parametric data) was used to evaluate differences in various clinical variables, laboratory values, and ESA treatment outcomes at different time points. For categorical variables, the chi-square test or Fisher’s exact test was applied. ROC analysis was conducted to assess the predictive performance of EPO and TGF-β1 staining, with the AUC and 95% CI calculated for each parameter. The optimal cut-off point was determined by maximizing the Youden Index. Finally, the Kaplan–Meier method was used to compare overall survival and disease-free survival in relation to various clinical factors. *P* < 0.05 was considered statistically significant for all analyses.

## Results

The study included 56 low and very low-risk MDS patients. Their demographic and clinical characteristics are summarized in Table 1. Among them, 33 were male and 23 were female, with a median age of 66.5 years (range: 34–88). At diagnosis, six patients had hypothyroidism, 28 had ischemic heart disease, six had type 2 diabetes mellitus, and seven had rheumatologic diseases. ESA treatment was administered to 45 patients (80.4%), while 11 (19.6%) did not receive it. During follow-up, 22 patients (39.3%) required G-CSF. Secondary AML developed in 10 patients (17.9%), including eight of the 22 (36.3%) who had used G-CSF. The mean hemoglobin level at diagnosis was 8.6 ± 1.2 g/dL in patients receiving ESA therapy and 9.2 ± 2.4 g/dL in those who did not. The median EPO level for all patients was 63 IU/L (range: 7.3–1760), while for those receiving ESA treatment, it was 58 IU/L (range: 7–624). Immunohistochemical analysis of bone marrow samples at diagnosis showed that only five patients (9.8%) exhibited weak SMAD-7 staining, while the remaining 51 (90.2%) showed no staining (Figure 2). For TGF-β1, 18 patients (32.1%) demonstrated moderate to strong staining, whereas 38 (67.9%) had weak (36/38) or no staining (2/38) (Figure 2). The median follow-up period was 27 months (range: 1.1–92.4). The median OS for all patients was 46.6 months (95% CI: 26.6–67.9), while the median PFS was 45.0 months (95% CI: 28.9–61.1). In total, 26 patients (46.4%) died, including four from AML transformation and 22 from non-cancer-related causes. Comparison of TGF-β1 staining before and after 2019 (including 2019) revealed no significant difference between the groups (*n* ═ 31 vs *n* ═ 25, *p* ═ 0.149). Cytogenetic analysis identified one patient with del (7q), one with trisomy 8, and one with monosomy 7. All three patients with chromosome 7 abnormalities showed moderate TGF-β1 staining, while the trisomy 8 patient had weak staining. Each of these three patients eventually progressed to AML during follow-up.

**Table 1:** Distribution of sociodemographic and clinical characteristics of the patients

**Variables**		***N***	**%**
Gender	Male	33	58.9
	Female	23	41.1
Age		Median (min–max) 67 (34–88)	
Hgb at diagnosis (gr/dL)	Patients receiving ESA treatment (mean ± SD) Patients not receiving ESA treatment (mean ± SD)	8.6 ± 1.2 9.2 ± 2.4	
EPO (IU/L)		63 (7.3–1760)	
*ESA given ESA not given*		58 (7–624) 500 (7.5–1760)	
IPSS-R	Very low risk	44	78.5
	Low risk	12	21.5
SMAD-7 staining	No staining	51	90.2
	Weak staining	5	9.8
TGF-β1 staining	No/Weak staining	38	67.9
	Moderate/Strong staining	18	32.1
ESA tx	Not given	11	19.6
	Given	45	80.4
Use of G-CSF	No	34	60.7
	Yes	22	39.3
Hypothyroidism	No	50	89.3
	Yes	6	10.7
Heart failure	No	28	50.0
	Yes	28	50.0
Type2 diabetes	No	50	89.3
	Yes	6	10.7
Rheumatological disease	No	49	87.5
	Yes	7	12.5
ESA tx 1st month response	No	17	37.8
	Yes	28	62.2
ESA tx 3rd month response	No	17	44.7
	Yes	21	55.3
Sec AML	No	46	82.1
	Yes	10	17.9

Hgb: Hemoglobin; Sec: Secondary; G-CSF: Granulocyte-colony stimulating factor; EPO: Erythropoietin; TGF-β1: Transforming growth factor beta 1; SMAD-7: Mothers against decapentaplegic homolog 7; ESA: Erythropoietin stimulating agent; AML: Acute myeloid leukemia; IPSS-R: Revised international prognostic scoring system.

**Figure 3.**
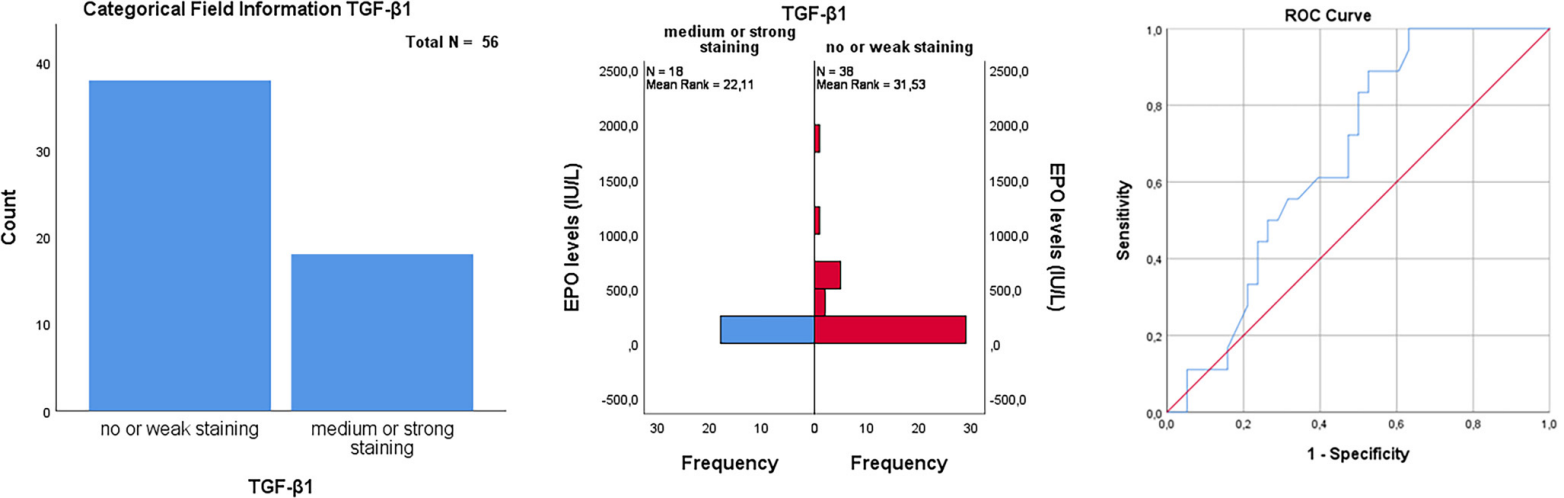
**TGF-β1 and EPO relationship**. Mann–Whitney *U*-test (*P* ═ 0.04). TGF- β1: Transforming growth factor beta 1; EPO: Erythropoietin.

**Figure 4.**
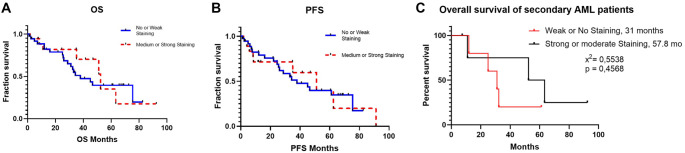
**Kaplan–Meier curves of overall survival and progression-free survival according to TGF-β1 status**. TGF-β1: Transforming growth factor beta 1.

When comparing TGF-β1 and SMAD-7 expression between patients receiving ESA therapy and those not receiving it, no statistically significant difference was found in SMAD-7 expression (*P* ═ 0.571). However, a significant difference was observed in TGF-β1 expression (*P* ═ 0.011). Moderate/strong TGF-β1 staining indicates that patients may be candidates for ESA therapy (Table 2). A comparison between TGF-β1 and EPO levels revealed a significant relationship between low EPO levels (AUC: 0.668, *P* ═ 0.044, cut-off: 58.5 IU/L) and moderate/strong TGF-β1 staining (*P* ═ 0.04) (Figure 3). However, in patients receiving ESA therapy, TGF-β1 staining did not significantly correlate with treatment response at either the first or third month (first month: *P* ═ 0.451; third month: *P* ═ 0.847) (Table 2). Additionally, when TGF-β1 staining was analyzed in patients receiving G-CSF treatment, no significant difference was found between groups (*P* ═ 1.000), indicating that TGF-β1 staining did not predict whether patients received G-CSF supplementation. When evaluated in relation to OS and PF, patients with moderate/strong TGF-β1 staining had higher median OS (51.0 vs 30.9 months) and PFS (51.0 vs 37.9 months) compared to those with weak/no staining. However, these differences were not statistically significant (OS: *P* ═ 0.689; PFS: *P* ═ 0.905) (Figure 4). Regarding transformation into secondary AML, moderate/strong TGF-β1 staining was observed more frequently in patients who developed secondary AML (27.8%) compared to those with weak/no staining (13.2%), but this difference was not statistically significant (*P* ═ 0.263). Furthermore, when comparing OS in secondary AML patients based on TGF-β1 staining, those with moderate/strong staining had longer survival (31 vs 57.8 months), though the difference was not statistically significant (*P* ═ 0.4568) (Figure 4). Lastly, we investigated whether patients’ comorbid conditions influenced ESA therapy response. No significant correlation was found between hypothyroidism, heart failure, type 2 diabetes mellitus, rheumatologic conditions, and ESA therapy response at either the first or third month (Table 3).

**Table 2:** Comparison of SMAD-7 and TGF-β1 staining according to ESA groups

	**ESA, at diagnosis**	
	**Not given** **(*n* ═ 11)**	**Given** **(*n* ═ 45)**	*P*
*SMAD-7 score, n (%)*			
No staining	11 (100.0)	40 (88.9)	0.571^a^
Weak staining	0 (0.0)	5 (11.1)	
*TGF-β1, n (%)*			
No/Weak staining	11 (100.0)	27 (60.0)	**0.011^a^**
Moderate/Strong staining	0 (0.0)	18 (40.0)	
	**ESA first month**		
	**No response** **(*n* ═ 17)**	**Response** **(*n* ═ 28)**	*P*
*SMAD-7 score, n (%)*			
No staining	15 (88.2)	25 (89.3)	0.914^a^
Weak staining	2 (11.8)	3 (10.7)	
*TGF-β1, n (%)*			
No/Weak staining	9 (52.9)	18 (64.3)	0.451^a^
Moderate/Strong staining	8 (47.1)	10 (35.7)	
	**ESA third month**		
	**No response** **(*n* ═ 17)**	**Response** **(*n* ═ 21)**	*P*
*SMAD-7 score, n (%)*			
No staining	15 (88.2)	18 (85.7)	0.819^a^
Weak staining	2 (11.8)	3 (14.3)	
*TGF-β1, n (%)*			
No/Weak staining	10 (58.8)	13 (61.9)	0.847^a^
Moderate/Strong staining	7 (41.2)	8 (38.1)	

a: Fisher’s exact test, *P* < 0.05 statistically significant. TGF-β1: Transforming growth factor beta 1; SMAD-7: Mothers against decapentaplegic homolog 7; ESA: Erythropoietin stimulating agent.

**Table 3:** The effect of patient comorbidities on ESA first and third month response

	**ESA first month**		
	**No response** **(*n* ═ 17)**	**Response** **(*n* ═ 28)**	*p*
*Hypothyroidism, n (%)*			
No	14 (82.4)	25 (89.3)	0.658^b^
Yes	3 (17.6)	3 (10.7)	
*Heart failure, n (%)*			
No	8 (47.1)	13 (46.4)	0.967^a^
Yes	9 (52.9)	15 (53.6)	
*Type 2 diabetes, n (%)*			
No	13 (76.5)	26 (92.9)	0.179^b^
Yes	4 (23.5)	2 (7.1)	
*Rheumatologic, n (%)*			
No	16 (94.1)	23 (82.1)	0.385^b^
Yes	1 (5.9)	5 (17.9)	
	**ESA third month**		
*Hypothyroidism, n (%)*			
No	16 (94.1)	18 (85.7)	0.653^b^
Yes	1 (5.9)	3 (14.3)	
*Heart failure, n (%)*			
No	7 (41.2)	10 (47.6)	0.691^a^
Yes	10 (58.8)	11 (52.4)	
*Type 2 diabetes, n (%)*			
No	13 (76.5)	19 (90.5)	0.378^b^
Yes	4 (23.5)	2 (9.5)	
*Rheumatologic, n (%)*			
No	15 (88.2)	18 (85.7)	0.604^b^
Yes	2 (11.8)	3 (14.3)	

a: Chi-square test; b: Fisher’s exact test, *P* < 0.05 statistically significant. ESA: Erythropoietin stimulating agent.

## Discussion

Due to the limited understanding of the etiology behind the ineffective hematopoiesis in MDS, developing effective therapies remains a complex and significant challenge. Addressing this issue is of utmost importance. In the present study, bone marrow SMAD-7 expression levels were found to be suppressed in patients with low-risk MDS at diagnosis, while TGF-β1 expression was moderate to strong in approximately one-third (32.1%) of patients. However, no correlation was observed between ESA treatment response and TGF-β1 expression when analyzed based on TGF-β1 levels. Notably, the most significant finding of our study was that moderate to strong TGF-β1 staining served as a predictor of ESA therapy response. These results are particularly important, as this is the first study to establish a relationship among TGF-β1, ESA treatment response, and EPO. Previous studies on SMAD-7 expression in MDS patients have reported similar findings. One study found that SMAD-7 gene expression was reduced by more than twofold in 83 of 89 patients compared to controls. Immunohistochemical analysis in the same study showed weak SMAD-7 staining in 75% of MDS patients, observed across all precursor cell lineages, including erythroid and myeloid (75% vs 9% in controls) [20]. Another study reported significantly lower SMAD-7 gene expression in MDS patients compared to healthy controls (log2 expression: 8.31 in controls vs 6.32 in MDS patient samples) [7]. In our study, immunohistochemical analysis showed weak SMAD-7 staining in only five of 56 patients, aligning with previous findings. This consistency with the literature reinforces the relevance of our observations.

A study examining the relationship between SMAD-7 and TGF-β1 in patients with low-risk MDS found that inhibiting SMAD-7 expression using a lentivirus significantly increased TGF-β1 sensitivity [20]. Another study demonstrated that inhibiting microRNA-21 abolished the inhibitory effect of TGF-β1 on erythroid cells via SMAD-7. The same study also showed that microRNA-21 inhibition led to increased SMAD-7 staining and decreased SMAD-2 staining—an indirect indicator of TGF-β1—on immunohistochemical analysis [7]. In the present study, TGF-β1 staining was observed in 54 patients, while two patients showed no staining. Among those with staining, 18 exhibited moderate to strong staining. These findings further support the relationship between SMAD-7 and TGF-β1, consistent with previous literature. ESAs remain the primary treatment for patients with low-risk MDS. These agents include recombinant EPO and darbepoietin. It is important to note that EPO levels vary among patients, and studies have shown that EPO levels can help predict ESA therapy response [22]. Generally, patients with EPO levels below 500 IU/L are more likely to respond to ESA therapy, with levels below 200 IU/L being associated with an even better response [22, 23]. In the present study, the median EPO level was 63 IU/L (range: 7.3–1760 IU/L). Notably, all patients receiving ESA therapy had EPO levels below 500 IU/L.A significant correlation was found between moderate to strong TGF-β1 staining and the administration of ESA therapy (*P* ═ 0.011). However, while TGF-β1 staining levels were predictive of ESA therapy administration, they did not predict treatment response. No significant difference was observed in first- or third-month treatment responses among ESA-treated patients in relation to TGF-β1 staining (first-month *P* ═ 0.451; third-month *P* ═ 0.847). This suggests that while TGF-β1 levels play a role in low-risk MDS, additional mechanisms within this signaling pathway influence treatment response. Recent research on sotatercept—an activin receptor IIA ligand trap—has shown promise. Like luspatercept, sotatercept targets the TGF-β pathway, offering potential new treatment strategies for patients with low-risk MDS [24].

Inhibition of the TGF-beta pathway has shown promising results in stimulating erythropoiesis and ameliorating anemia in patients with low-risk MDS. These findings underscore the central role of the TGF-beta pathway in MDS pathogenesis and provide a compelling rationale for developing novel therapeutic approaches targeting this pathway. Notably, molecules such as elritercept—a recombinant fusion protein consisting of a human IgG1 Fc linked to a modified activin receptor type IIA extracellular domain—act as soluble ligand traps, inhibiting activin A and other specific TGF-β superfamily ligands, including activin B, growth differentiation factor 8 (GDF-8), and GDF-11. *In vivo* and *in vitro* studies have shown that RKER-050, a research form of elritercept, promotes differentiation across both early and terminal stages of erythropoiesis and thrombopoiesis, supporting a distinct pharmacological profile. Preclinical studies further demonstrated that RKER-050 increased both RBC and platelet production in healthy and diseased rodent models [14]. Additionally, combining luspatercept with ESAs has shown promise in enhancing treatment responses in certain MDS patients [25]. Phase 1 and 2 studies on TP-0184, an activin-receptor-like kinase 5 inhibitor that acts through the TGF-β/SMAD-2/3 pathway, are also ongoing [26]. Another notable finding in the present study was the relationship between TGF-β1 staining levels and AML transformation. Although not statistically significant (*P* ═ 0.263), transformation into secondary AML was more frequent in patients with moderate/strong staining (27.8%) compared to those with weak/no staining (13.2%). Other studies have reported a negative correlation between TGF-β receptor expression and AML patient survival, with shorter survival observed in patients with high expression levels [27, 28]. However, in our study, survival was better in patients with moderate/high TGF-β1 expression, suggesting that additional factors and surface markers may influence survival and AML transformation. One such factor is G-CSF. In the present study, 8 of 22 patients who received G-CSF progressed to AML. A systematic review by Lyman et al. [29] found that while G-CSF use was associated with increased AML transformation, it was also linked to improved survival due to immune system enhancement. Genetics is likely one of the most critical factors influencing AML transformation. According to recent MDS classification guidelines, genetic risk stratification supersedes other prognostic factors in lower-risk MDS [30, 31]. In our study, three patients had cytogenetic abnormalities involving chromosomes 7 and 8. Two of these three patients had moderate TGF-β1 staining, and all three progressed to AML. Interestingly, a study on low-risk MDS patients found that isolated del(7q) is associated with good overall survival [32]. Furthermore, a phase 3 study on darbepoetin in low-risk MDS (including lower-risk cytogenetics) concluded that darbepoetin does not increase the risk of AML transformation [33]. A French study evaluating the impact of ESA treatment on low-risk genetic factors in MDS patients found that ESA-treated patients had better survival outcomes (HR for death ═ 0.43, 95% CI 0.25–0.72) [34].

However, it is important to acknowledge that research in this field is still in its early stages, and further investigation is warranted. Specifically, a more in-depth understanding of the efficacy of TGF-beta inhibition across different MDS subtypes, the roles of various SMAD proteins, and the interactions of this pathway with other signaling cascades is needed. In the future, identifying TGF-beta pathway biomarkers could help predict patient responses to treatment, facilitating the development of personalized therapeutic strategies. Our study contributes to the advancement of more effective and safer treatment options for patients with low-risk MDS. Notably, our findings suggest that TGF-β1 staining should be considered alongside other prognostic markers for AML transformation, despite previous studies highlighting the predictive value of genetic factors, G-CSF, and ESA treatment. However, this study has several limitations, including the small sample size, its retrospective design, the absence of next-generation sequencing (including SF3B1) in the genetic evaluation, and the lack of ringed sideroblast data.

## Conclusion

These findings suggest that additional TGF-β/SMAD pathway-related parameters should be considered, as TGF-β1 alone is ineffective in determining ESA treatment response. The decision to initiate ESA treatment may be influenced by strong TGF-β1 staining just as much as by EPO levels. However, multicenter studies with larger patient populations are needed to validate these results and further explore the role of the TGF-β signaling pathway.

## Data Availability

Data available on request due to privacy/ethical restrictions.
